# Immunogenetic Aspects of Sarcopenic Obesity

**DOI:** 10.3390/genes15020206

**Published:** 2024-02-05

**Authors:** Łukasz Mazurkiewicz, Krystian Czernikiewicz, Bogna Grygiel-Górniak

**Affiliations:** Department of Rheumatology, Rehabilitation and Internal Diseases, Poznan University of Medical Sciences, 61-701 Poznan, Poland

**Keywords:** sarcopenic obesity, genetic background, immunogenetics aspects

## Abstract

Sarcopenic obesity (SO) is a combination of obesity and sarcopenia, with diagnostic criteria defined as impaired skeletal muscle function and altered body composition (e.g., increased fat mass and reduced muscle mass). The mechanism of SO is not yet perfectly understood; however, the pathogenesis includes aging and its complications, chronic inflammation, insulin resistance (IR), and hormonal changes. Genetic background is apparent in the pathogenesis of isolated obesity, which is most often polygenic and is characterized by the additive effect of various genetic factors. The genetic etiology has not been strictly established in SO. Still, many data confirm the existence of pathogenic gene variants, e.g., Fat Mass and Obesity Associated Gene (*FTO*), beta-2-adrenergic receptor (*ADRB2*) gene, melanocortin-4 receptor (*MC4R*) and others with obesity. The literature on the role of these genes is scarce, and their role has not yet been thoroughly established. On the other hand, the involvement of systemic inflammation due to increased adipose tissue in SO plays a significant role in its pathophysiology through the synthesis of various cytokines such as monocyte chemoattractant protein-1 (MCP-1), IL-1Ra, IL-15, adiponectin or CRP. The lack of anti-inflammatory cytokine (e.g., IL-15) can increase SO risk, but further studies are needed to evaluate the exact mechanisms of implications of various cytokines in SO individuals. This manuscript analyses various immunogenetic and non-genetic factors and summarizes the recent findings on immunogenetics potentially impacting SO development.

## 1. Introduction

Sarcopenic obesity (SO) involves both the gain of fat mass (obesity), and sarcopenia is defined as low muscle strength resulting from low muscle quantity or quality [1,2]. The previous lack of universal diagnostic criteria made it difficult to estimate SO’s prevalence and to plan treatment strategies [1]. 

The published standards by the European Society for Clinical Nutrition and Metabolism (ESPEN) and the European Association for the Study of Obesity (EASO) allow us to gather more information about this condition, which is typical for an aging society [1]. The current definition of SO includes the coexistence of obesity and sarcopenia, and the diagnostic criteria are based on impaired skeletal muscle function and altered body composition–like increased fat mass and reduced muscle mass [1]. Two recent meta-analyses estimated the prevalence of SO as between 9% and 11%; however, the lack of universal SO definition and the incoherence of definitions used to define obesity or sarcopenia separately, make the SO prevalence still inconclusive and needing further investigation [3,4,5]. The prevalence of SO increases with age and is associated with accumulation of chronic diseases—for example fractures, cardiovascular diseases or cancer [1,6,7]. Higher all-cause mortality rate in women suffering from SO was observed and a higher risk of death within 10 years in 75-years-old SO women was reported [7,8]. Pathogenesis of SO includes aging, strictly associated with physical inactivity and often malnutrition, as well as chronic inflammation, insulin resistance (IR), and hormonal changes [9]. The mechanism of SO is not yet perfectly understood; however, it is a complex condition and is characterized by both obesity and sarcopenia, with overlapping features [10]. The aim of this review is to indicate the multifactorial and immunogenetic nature of SO, which involves both genetic predisposition and immune system involvement in the pathophysiology of the disease.

## 2. Materials and Methods

**Figure 1:**
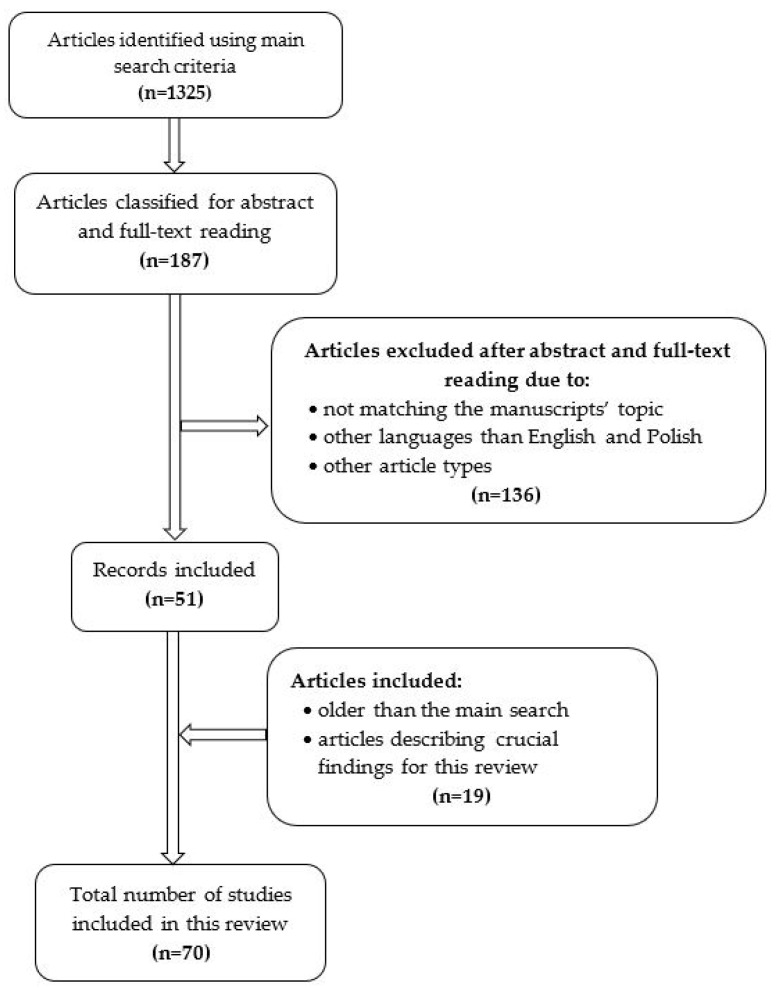
Graphical presentation of the search strategy used in this review.

Publicly available databases on medical sciences, like PubMed or Google Scholar, were searched through to identify records published between 2014–2023. The search strategy included main words—sarcopenia and obesity—and other keywords related to the pathogenesis of obesity, sarcopenia, and SO, reaching the major areas such as prevalence, mechanisms, immunology, and genetics. Various connections between keywords were made, combined with obesity or sarcopenia, using the conjunction words like AND or OR. All article types were considered if only the main search criteria were met. At first, titles were screened for accuracy. Articles that qualified for evaluation were analyzed through the abstract and the whole text. Manuscripts in English and Polish language were included in this analysis. Exceptionally, besides the search criteria, additional articles from the research field describing crucial findings were included regardless of the publication year. The search strategy is presented in Figure 1.

## 3. Obesity and Sarcopenia—What Came First: The Egg or the Chicken?

Obesity is a complex multifactorial disease characterized by accumulated excess body fat, negatively affecting health [11]. Obesity in most of the global population is defined as a BMI value equal to or over 30 kg/m^2^, while for Asians, it is equal to or above 25 kg/m^2^ [11,12]. In turn, sarcopenia is defined as loss of muscle mass and strength or physical function, which naturally occurs in aging. This condition is typical for a geriatric population, and its prevalence increases with age [2,13,14]. Sarcopenia is related to frailty and multi-morbidity and is often observed in users of residential aged care (nursing home) services [15,16]. Since sarcopenia is a highly prevalent condition in older people and is related to disability, hospitalization, and death, quick diagnosis and adequate prevention and treatment should be implemented [17].

The definition of sarcopenia has developed over the last two decades. The original meaning of this condition only included reduced muscle mass. With modern anthropometric techniques and imaging capabilities, the definition of sarcopenia has evolved (Table 1). 

**Figure 2:**
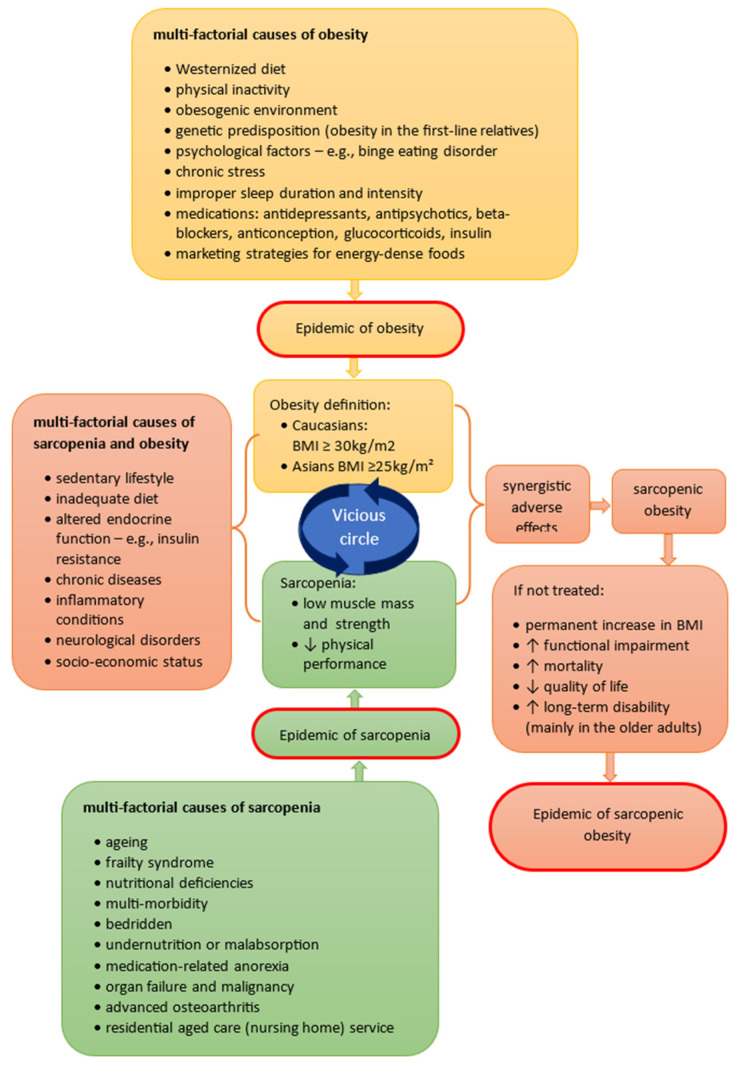
Multi-factorial causes of obesity, sarcopenia and SO.

New tools and methods increase the possibility of sarcopenia diagnosis more precisely, permitting comparisons between different social groups [22] (Table 2). This was a milestone that opened up the possibility of diagnosing sarcopenia in younger age groups and, consequently, increased scientific curiosity about which risk factors are related to its development (Figure 2). Since then, sarcopenia has also been reported in young people who had low physical activity associated with long-term immobilization [2,23,24]. However, determining the prevalence of sarcopenia in the young population is challenging; however, based on the evidence, more than one subject in every ten young adults of most ethnicities is estimated to have sarcopenia. 

Conversely to obesity assessment, defining sarcopenia is difficult because of technical problems in the accurate evaluation of muscle mass and muscle quality [25,26,27,28]. In the literature, there is a broad discussion about the techniques of muscle mass and strength estimation; however, recent data confirm that the estimation of muscle strength better than mass predicts adverse outcomes of sarcopenia [24,29]. Unfortunately, there is no international consensus on a definition of sarcopenia. Many reports attempt to describe this phenomenon, as exemplified by consensuses published by the European Working Group on Sarcopenia in Older People (EWGSOP1 and EWGSOP2) [2,13], the Foundation for the National Institutes of Health (FNIH) [20] or Australian and New Zealand Society for Sarcopenia and Frailty Research (ANZSSFR) [21] (Table 1). Therefore, there are many delineations in sarcopenia definitions, although most are convergent and consider low muscle mass and low muscle strength or performance as the exponent of sarcopenia. Uncertainties in sarcopenia diagnosis are also related to the evaluation methods, which differ in how they assess the quantity and quality of muscles and propose different points (Table 2). The EWGSOP suggests that muscle mass should be expressed relative to height, while the FNIH recommends adjustment by BMI. Such discrepancies contribute to inconsistencies in estimating the prevalence of sarcopenia and cause difficulties in comparison of various populations [30,31,32].

**Table 2:** Methods of muscle mass estimation.

Muscle Mass Estimation and Cut-Off Points in Sarcopenic Patients		
Method	Criteria	Reference
Anthropometric	Reference population: Rosetta study [18]—a population-based survey of 883 elderly Hispanic and non-Hispanic white men and women living in New Mexico ALM/ht^2^ M ≤ 7.26 kg/m^2^ F ≤ 5.45 k/m^2^	[18,19]
Dual X-ray absorptiometry—DXA	reference population: Rosetta study [18] **Sarcopenia Index** ALM/ht^2^ M ≤ 7.26 kg/m^2^ F ≤ 5.45 k/m^2^	[33,34,35]
	STUDY: Health ABC—the Health Aging and Body Composition **Sarcopenia index**: ALM/ht^2^ M ≤ 7.23 kg/m^2^ F ≤ 5.67 kg/m^2^	[36]
	DXA—less than the twentieth percentile of healthy adults **Sarcopenia index**: ALM/ht^2^ M ≤ 7.23 kg/m^2^ F ≤ 5.67 kg/m^2^	[17]
Bioelectrical impedance—BIA	STUDY: NHANES III—the Third National Health and Nutrition Examination Survey Sarcopenia definition: ratio of muscle mass/total body mass M ≤ 31.5% F ≤ 22.1%	[37]
	Study population: subjects who attended a 1988–1992 Rancho Bernardo Study clinic Sarcopenia definition: the value of FFM of > or =2.0 standard deviations below the gender-specific mean of a young reference population (Mean FFM was 43.5 kg for women and 61.7 kg for men.) **Sarcopenia index**—Total lean mass/ht^2^ M ≤ 8.50 kg/m^2^ F ≤ 5.75 kg/m^2^	[38]
	STUDY: NHANES III—the Third National Health and Nutrition Examination Survey **Sarcopenia definition**: total muscle mass/ht^2^: M ≤ 8.50 kg/m^2^ F ≤ 5.75 kg/m^2^	[39]
	STUDY: Cardiovascular Health Study **Sarcopenia definition**: fat-free mass: M ≤ 47.9 kg F ≤ 34.7 kg	[40]
**Estimation of cut-off points for SO**		
dual X-ray absorptiometry—DXA	Analysis of two definitions of SO: **New Mexico Elder Health Survey** Obesity definition: BMI ≥ 30 kg/m^2^ Sarcopenia assessment: ALM divided by height squared (M < 7.23 kg/m^2^ and F < 5.67 kg/m^2^) **Health ABC study** Obesity definition: BMI ≥ 30 kg/m^2^ ALM divided by height and fat mass DXA—lowest twentieth percentile of residuals (sex-specific) Sarcopenia, defined by these two definitions, has differential prevalence rates by obesity status, which underlines the necessity of fat mass assessment in evaluating sarcopenia.	[36]
	**New Mexico Elder Health Survey:** Obesity definition: body fat: M ≥ 28% and F ≥ 40% Sarcopenia assessment: ALM divided by height squared DXA: M < 7.26 kg/m^2^ and F < 5.45 kg/m^2^	[41]
	**Nutrition as a Determinant of Successful Aging study** Obesity definition: Body fat (M ≥28% and F ≥35%) Sarcopenia assessment: ALM divided by height squared DXA: M < 8.51 kg/m^2^ and F < 6.29 kg/m^2^	[42]

ALM = appendicular lean mass; ALM/ht^2^—appendicular lean mass divided by height squared; DXA—dual X-ray absorptiometry; FFM—free fat mass; M—male; F—female.

In the literature SO is defined as excess fat mass and sarcopenia [43]. Nevertheless, obesity is a separate syndrome, and not each type of obesity is related to sarcopenia. Since body mass loss results in a decrease in both fat mass and lean body mass, it is possible that decreasing weight in obese older persons could worsen the age-related loss of muscle mass and increase sarcopenia [43]. Furthermore, there is a synergistic adverse effect of sarcopenia and obesity, particularly in older populations, which results in an increasing prevalence of SO [44]. It is also worth underlining that synergistic problems of sarcopenia and obesity lead to impairments in instrumental activities of daily living [41]. 

The definitions of SO have been proposed by two associations: the European Society for Clinical Nutrition and Metabolism (ESPEN) and the European Association for the Study of Obesity (EASO). According to the initiative of these societies, SO is defined as the co-existence of excess adiposity and low muscle mass/function. The diagnosis of SO should be considered in at-risk individuals with elevated BMI or waist circumference and markers of low skeletal muscle mass and function. It is extremely important to determine associated comorbidities. Diagnosis of SO should be confirmed by decreased skeletal muscle function and improper body composition (excess adiposity and low skeletal muscle mass). Each SO patient should have been screened for possible related-body composition clinical complications [1]. 

The synergistic risk factors of the development of obesity and sarcopenia at a young age are similar in adults and older people and include physical inactivity, a not-well-balanced diet (e.g., a pro-inflammatory diet deficient in antioxidant vitamins A and E), metabolic syndrome, vitamin D deficiency, inherent and perinatal factors, gut microbiota dysbiosis, endocrinopathy, malignancy, and other inflammatory disorders [23]. The similar pathological pathways suggest the relationship between both obesity and sarcopenia, and the development of one condition may increase the risk of another one; however, this hypothesis requires future studies. It is worthy to underline that the phenotype of sarcopenia has many contributing causes unrelated to aging [45,46]. Such implications indicate that the well-described obesity-related genes may also influence the sarcopenic genotype. Such implications suggest that well-described obesity-related genes may be valuable information for searching for the causes of SO and enabling individualized therapy planning.

## 4. Immune Involvement in Sarcopenic Obesity

### 4.1. Pro-Inflammatory Cytokine Synthesis and Their Relation to Sarcopenic Obesity

Increased adipose tissue causes systemic inflammation, which plays a major role in the pathophysiology of SO due to the effect on skeletal muscles and the decrease in oxidative capacity [47]. Both obesity and sarcopenia are strictly connected with chronic inflammation [48,49]. Obesity leads to increased adipocyte macrophage infiltration, resulting in further adipokine secretion. Adipose tissues change their physiological activity and release excess free fatty acids, reactive oxygen species, and pro-inflammatory cytokines [50]. Ectopic fat deposition results in organelle damage and dysfunction, causing IR [50]. Additionally, both the production and efficiency of insulin decrease with age. Additionally, insulin low-grade inflammation and cytokine synthesis decrease insulin sensitivity. With aging, obesity associated with insulin resistance leads to muscle catabolism, reduction in muscle strength, and, finally, SO [9].

MCP-1 plays a major role in the pathogenesis of obesity [51]. A study by Huber et al. demonstrated elevated gene expression of CC chemokines (a family of chemokines that attract inflammatory mononuclear cells to the inflammation sites) and their receptors, including MCP-1 and C-C motif chemokine receptor 2 (CCR2) in subcutaneous and visceral adipose tissue in obese patients [52]. MCP-1 helps the CCR2-positive monocytes to recruit and infiltrate the adipose tissues, which are transformed into M1 macrophages [50,53,54]. M1 macrophages are responsible for synthesizing TNF-α, IL-6, and MCP-1 [54]. This results in further cytokine expression [50]. IL-6 levels, IL-1 receptor antagonist (IL-1Ra), and soluble IL-6 receptor levels are elevated, which is crucial in SO development [49]. 

Physiologically, muscle-produced IL-6 positively impacts muscle regeneration and hypertrophy; however, in persistent inflammatory conditions, like the obese state, it acts as a pro-inflammatory, activating the *MAP3K8* expression, further influencing NF-kB and JNK transcription factors, leading to IR [10,55,56]. Excessive secretion of IL-6 is observed in adiposity. Moreover, the *IL-6*-174G>C polymorphism in the *IL-6* promoter region significantly increases the risk of obesity [57]. Other *IL-6* gene polymorphisms, such as rs1800795(G/C) and rs1800796(G/C), are the risk factors for obesity and are considered potential new targets for drug inventions in obesity treatment [58]. 

Another cytokine secreted in obesity is IL-1Ra. This cytokine is a natural antagonist in the IL-1 family and reveals a counterregulatory (anti-inflammatory) function. The disproportions between pro-inflammatory IL-1 and anti-inflammatory IL-1Ra lead to the type 1 diabetes mellitus (T1DM) [59]. 

The plasma level of IL-15 decreases in sarcopenia [10]. The function of this cytokine impacts the anabolic effect on muscle growth [56]. It has been documented that the older people (above 95 years of age) living independently have greater levels of IL-15 than the two control groups—those between 30–59 years of age and 60–89 years of age. Thus, IL-15 has a positive impact on aging [60]. This cytokine stimulates the upregulation of the pro-oxidative PPARδ and SIRT1 benefit against obesity and IR [61]. Thus, IL-15 can be one of the beneficial factors during sarcopenia treatment related to advanced aging [10].

Adiponectin, a crucial player in obesity prevention, is responsible for insulin activity and regulating further pro- and anti-inflammatory cytokines. It enhances the production of IL-10 and IL-1Ra and reduces the amount of TNF-α [55,62,63]. The plasma concentration of adiponectin negatively correlates with body fat mass [63]. Another adipocytokine is leptin, which reveals pro-inflammatory activity and stimulates the production of IL-6, IL-12, and TNF-α. Its plasma concentration is positively correlated with body fat mass [62,63]. High levels of TNF-α and IL-6 are observed in sarcopenia and muscle weakness [56].

In summary, many cytokines participate in obesity pathogenesis. Some of them (like IL-6) have anti-inflammatory activity in the physiological state, while they gain pro-inflammatory properties in the obesity state [10]. The lack of anti-inflammatory cytokine (e.g., IL-15) can be one of the mechanisms leading to increased SO risk. Further investigations are needed to evaluate the exact mechanisms of various cytokines in SO individuals.

### 4.2. Hormonal Factors Related to Immune Response and Genetic Background

SO has been associated with hormonal factors. With age, the functioning of the human body, including its humoral aspects, undergoes changes that affect hormonal regulation. These systemic changes can be an underlying factor leading to the development of sarcopenia and SO [64]. 

Sex-specific hormonal changes characteristic of older adults are associated with SO [64,65]. Low estrogen levels after menopause are related to increased body weight. Also, the localization of adipose tissue is altered from subcutaneous to visceral [65]. Low estrogen levels are also associated with bone mass reduction [64]. In males, the testosterone level stimulates muscle synthesis [66]. In older males, decreasing testosterone levels correlate with lower muscle strength, muscle mass, poor physical performance, and SO [64,65,67]. 

Not only sex hormones, but also cortisol, growth hormone, insulin, and insulin-like hormones play a role in SO pathophysiology [64,66,68]. Insulin resistance and inflammation are present due to changes in signaling in hormonal pathways, leading to the elevation of cytokines and causing oxidative stress [66]. Insulin also increases muscle mass. This fact explains why patients with diabetes develop sarcopenia more rapidly [64]. 

Ghrelin is a hormone produced in the stomach, precisely in its funds. It is most notable for its stimulatory effect on growth hormone release, food intake increase, and regulation of fat deposition [69]. Ghrelin, ghrelin analogs, growth hormone, and insulin growth factor-1 (IGF-1) are reported to increase muscle mass but not muscle strength [64]. Thus, the decreased ghrelin level can influence the risk of SO. Another factor participating in muscle condition is myostatin, which acts as a regulator for skeletal muscle growth. Its main effects are muscle atrophy and cachexia [70]. Low myostatin levels are associated with muscle development [64]. Thus, anti-myostatin antibodies can used for SO therapy [68].

Due to the role of hormones in the development of SO, experimental hormonal therapies have been undertaken to treat SO. Recent reports show the experimental attempts to use growth hormone, growth hormone-releasing hormone analogs, and hormone replacement therapy (HRT) [68]. In postmenopausal women, HRT increases muscle strength (especially estrogen use) [64].

### 4.3. Infections during Childhood and the Risk of Obesity

Obesity results from the interaction between genetic, environmental factors, and diet [71]. Unfortunately, childhood infections treated using antibiotics are related to microbial resistance to available agents [72]. Thus, appropriate management of antibiotic therapy is of first importance to prevent potential drug resistance, but that is an important modifiable risk factor for obesity during childhood exposure [71,73]. The crucial criteria include the first six months after delivery and the repeated use of more than one antibiotic during this period [73]. Antibiotic use influences gut microbiota composition and maturation and changes the metabolism path of carbohydrates to short-chain fatty acids [74,75]. Another hypothesis is the disturbed mitochondrial function, which leads to the reduction of mitofusin 2 (Mfn2) [71,76]. A decrease in the amount of Mfn2, which plays a role in obesity development, could have an impact on the epidemic of childhood obesity worldwide [71,73]. 

An inflammatory marker implicated in SO is C-reactive protein (CRP), and its relationship with SO in specific populations was observed [47,77]. CRP levels, as well as IL-6 levels, increase with age. Fortunately, increased physical activity reduces the CRP level [78]. CRP may also be a mortality predictor, especially in older patients unrelated to SO [79].

Ohler and Braddock suggest that infections are stronger risk factors for childhood obesity than the use of antibiotics [80]. Some of the adenoviruses increase adiposity in animals and affect obesity in humans. For example, a protein of Ad-36—namely the early 4 open reading frame 1 (E4-ORF1)—is necessary for causing acute adipogenic effects [81,82]. One of the most important mechanisms is that the virus activates PPAR*γ*, leading to increased adiposity by committing adult stem cells into adipocytes [82,83]. Another important protein necessary for maintaining Ad-36-induced obesity is monocyte chemoattractant protein-1 (MCP-1) [82]. Another example of viral infections affecting obesity are those caused by herpesviridae—especially CMV, being lipogenic and affecting various parameters of metabolic syndrome, like blood pressure after infection [82]. 

### 4.4. The Role of Maternal HLA in Sarcopenic Obesity

The human leukocyte antigen (HLAs) complex mediates a chronic inflammatory pathway. It is widely known to be associated with specific autoimmune diseases, including spondyloarthritis, Behçet’s disease, or multiple sclerosis [84]. A study concerning older adults with autoimmune diseases revealed a strong association between sarcopenia and autoimmune disease. However, investigating various HLA carriers with no autoimmune disease diagnosis unveiled an association of various HLA types, like HLA-DQA1*03:01 and HLA-DRB4*01:03, with sarcopenia. Additionally, some of them were more prevalent in women [84]. A strong association with muscle weakness was also found near the *HLA-DQA1* gene, known to be a rheumatoid entity [85]. Single nucleotide polymorphisms associated with SO were also found in genes related to immune surveillance: *HLA-DRB1* and *HLA-DRB5* [67]. *HLA-DQB1-AS1* seems to be associated with hand grip strength and is one of the genes that need further study [86].

### 4.5. The Link between Genetic Background and Inflammation in Sarcopenic Obesity

Though the macrophage population is a mix of M1 and M2 macrophages, the larger degree of obesity is associated with more accumulated adipose tissue macrophages and their activation, which is triggered by various inflammatory mediators—like CCR2—chemokine leading to organizing into an inflammatory M1 phenotype [87]. FTO plays a positive role in macrophage activation. However, *FTO* is downregulated in both subtypes but promotes polarization in both groups, which is essential for maintaining both phenotypes [88]. Another study demonstrated that FTO is a transcriptional suppressor of IFN-stimulated genes by suppressing STAT3 transcription factor activation. This path also refers to some genes encoding pro-inflammatory factors [89]. STAT3 itself was reported not only to both promote and inhibit oncogenesis in various mechanisms but also to have an impact on immune suppression and inflammation [90]. For example, it enhances the production of IL-10, which suppresses the production of pro-inflammatory cytokines [90]. A study by Dubey et al. demonstrated that in mice models, increased methylation of m6A-RNA and a decrease in *FTO* expression in the myocardium led to an increase in the expression of inflammatory myocardial cytokine genes—like *IL-6*, *TNF-α*, and *IL-1β* [91]. 

The expression of *PPARγ* is recently described to be essential in the differentiation processes of various immune cells—like macrophages or dendritic cells [92]. Like FTO, PPARγ plays a role in the polarization process of macrophages, favoring the alternative M2 phenotype [93].

**Figure 3:**
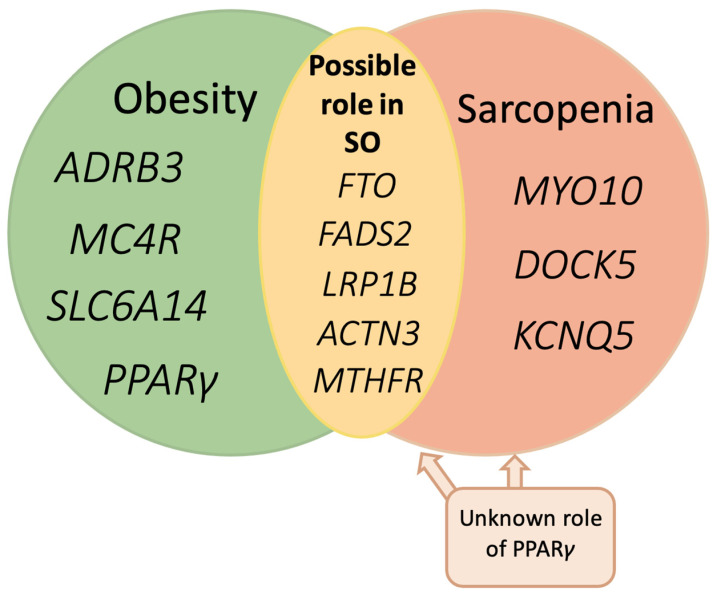
Genes related to obesity and sarcopenia (green—confirmed obesity-related gene polymorphisms; red—sarcopenia-related gene polymorphisms; yellow—probable gene polymorphisms in SO). The hypothesis concerns evidence from studies where the role of various selected polymorphisms is reported in the context of obesity and sarcopenia.

Specific *MTHFR* SNPs have been linked to both sarcopenia and obesity (Figure 3), leading to a hypothesis that these polymorphisms might play a role in SO. An *MTHFR* polymorphism has been associated with elevated IL-6 levels in a study by Araki et al. [94], suggesting a potential role of *MTHFR* polymorphism in pro-inflammatory cytokine secretion. An *MTHFR* polymorphism (rs1803311) has been positively linked to higher homocysteine levels, which promotes the elevation of pro-inflammatory markers such as TNF-α, IL-6, and IL-1β. Building on the established connection between folate levels and pro-inflammatory markers, it has been observed that supplementing folate through natural foods for an eight-week period resulted in a reduction of inflammatory markers and homocysteine levels [95].

*IL-6* polymorphisms have been linked to obesity in numerous studies. Researchers place *IL-6* polymorphisms, such as rs1800795 and rs1800796, as risk factors for obesity [58,96]. It is worth mentioning that some SNPs (rs1800797) are associated with a reduced risk of obesity [96]. 

*Interleukin 1* gene family has also been associated with the risk of obesity. As shown in the study by Melo et al., there has been a positive link between eleven variants of the *IL1B* gene and an increased risk of obesity in children [97]. Research conducted by Maculewicz et al. showed the involvement of *IL-1* family genes in obesity but emphasized that interactions between different polymorphisms should be sought [98].

Overall, the evidence on the correlation of gene polymorphisms (as depicted in Figure 3) and pro-inflammatory cytokine secretion is relatively scarce, leading to the conclusion that this should be a subject for further investigation.

## 5. Genetic Aspects of Sarcopenic Obesity

### 5.1. Genetic Susceptibility to Sarcopenic Obesity

Genetic factors play a major role in the pathogenesis of obesity. The cause of obesity is most often polygenic and is characterized by the additive effect of various genetic factors [99,100]. Genome Wide Association Studies (GWAS) are widely used to estimate genetic risk markers [101]. The difference between monogenic and polygenic obesity does not concern particular genes—the majority of the same genes play a role in both types of obesity. However, in polygenic obesity, the dysfunction of protein synthesis varies [102]. Obesity predisposition leads further to a pro-inflammatory state, resulting in the secretion of cytokines.

The identified genetic variants affecting the body mass are being used to make a polygenic risk score (PRO) to more accurately calculate overall genetic risks for obesity, simultaneously taking into account multiple gene variants [103,104]. For example, Tan and Mitra discovered higher odds of obesity in the third tertile of PRO regarding Fat Mass and Obesity Associated Gene (*FTO*) and beta-2-adrenergic receptor (*ADRB2*) gene variants [104]. Another study noticed the synergistic impact of other genes such as peroxisome proliferator-activated receptor gamma (*PPARγ*), *FTO*, and melanocortin-4 receptor (*MC4R*)—in the predisposition to overweight and obesity [105]. 

FTO initiates the onset of DM2 by increasing BMI value [100,106]. Its function is probably to regulate appetite and energy expenditure, as the highest levels are expressed in the hypothalamic regions [106,107]. It encodes a demethylase, being a family of AlkB family—ALKBH9 [107]. The main substrate is N6-methyladenosine (m6A), which plays a role in post-transcriptional regulatory processes [107]. An often studied SNP is rs9939609—homozygous for this variant in adults has a 1.67-fold increased risk of obesity [107]. Park and Choi found this dependence, especially in women [108]. Studies demonstrate that *FTO* SNPs may also be associated with lean mass index (LMI) and sarcopenia [109]. *FTO* rs9939609 AA homozygotes have been related to the risk for sarcopenia in older women [110]. These findings indicate this variant as a possible major factor in SO; however, to our knowledge, it is still unknown if *FTO* rs9939609 AA homozygotes are related to SO [109,110]. The same authors described endothelial nitric oxide synthetase gene—*NOS3* (rs1799983 GG genotype) to increase the sarcopenia risk almost twofold. It is postulated that this genotype makes the nitric oxide effect on muscles less effective, which explains the GG genotype’s potential complicity in sarcopenia [110].

Another genetic factor that may influence SO development is *MC4R* gene. This gene mutation is a flagship gene determining monogenic obesity [99]. Leptin, together with neuropeptide Y (NPY) and agouti-related peptide (AgRP), inhibits the appetite-stimulating system and activates the *MC4R* pathway, regulating hunger [111,112]. Most discovered *MC4R* mutations lead to a decrease in their function and result in obesity [99]. The melanocyte-stimulating hormone (MSH), a proopiomelanocortin (POMC) derived hormone, is stimulated by leptin, which helps reduce food intake. The POMC deficiency is rare [112]. MC4R agonist—setmelanotide is a promising agent in MC4R deficiency treatment, leading to significant weight loss [111,112]. To our knowledge, this genetic factor is not described in sarcopenia or SO. The rs17782313 T-allele reduces the incidence of lipid metabolism disorders and protects against metabolic disorders in Polish postmenopausal women [113]. A Chinese study confirms that the *MC4R* rs17782313 C/C genotype is associated with higher triglyceride (TG) levels in older Chinese women [114].

The beta-3 adrenergic receptor (*ADRB3*) gene regulates thermogenesis, and its expression is limited in the obese population [115]. Specific polymorphism of *ADRB3* gene—Trp64Arg impacts adipokines and lipid levels (mainly LDL-cholesterol) and is associated with obesity [116,117]. SO is regulated by adipose tissue and skeletal muscle amount ratio, which changes during aging, and in this process, *ADRB3* gene variants seem to play a crucial role. The function of this gene increases with age when adipose inflammation leads to the redistribution of fat to the intra-abdominal area and fatty infiltrations in skeletal muscles, resulting in decreased overall strength and functionality. Therefore, various metabolic alterations are observed, including insulin resistance. The expression of *ADRB3* is regulated by the diet. The study of animal models has shown that 3-week supplementation with a phenolic-rich olive leaf extract (100 mg/kg to Wistar rats orally) attenuated the aging-induced alterations in body composition and insulin resistance. Olive leaf extract treatment downregulated the expression of *ADRB3* and attenuated the aging-induced changes in the mRNA levels of insulin receptor and PPAR-γ [118]. 

*ADRB3* gene changes are observed in human studies. In non-obese adolescents, the less frequent allele (Trp64Arg and Arg64Arg) is related to higher LDL-cholesterol levels and lower maximal fat oxidation rates when compared with non-carriers (Trp64Trp) [117]. With age, the role of this *ADRB3* genotype increases, which reflects the tendency for increased worldwide obesity prevalence in an aging society. In the study of women with breast cancer, it was observed that homozygous individuals carrying the *ADRB3* wild-type allele exhibited significantly higher mean visceral fat levels compared to those with the variant allele, indicating a greater degree of obesity [119]. Overweight and obese women with Trp64Trp of *ADRB* gene polymorphism and normal TG levels are mainly normoglycemic, indicating this polymorphism’s beneficial influence on metabolic parameters [120]. Conversely, Arg64/X polymorphism of the *ADRB3* gene is related to dyslipidemia, particularly if nutrient intake is high which has proatherogenic effect due to overconsumption of fat, arachidic acid and overall high energy intake [121]. A similar relation is presented in the de Luis DA et al. study, which shows that obese subjects with the Arg64/X allele are predisposed to lipid disorders (higher TC, LDL, and TG) [122]. Not only in Caucasian populations [123] but also in Asian entities [124], the Arg64 allele is associated with obesity development. 

Nevertheless, epigenetic changes influencing the *ADRB3* gene are also crucial. DNA hypermethylation has been discovered to be related to being overweight, obese, and having a higher waist-hip ratio [115]. Thus, specific *ADRB3* gene polymorphism and epigenetic changes may impact sarcopenic development; however, data on the genetic involvement of *ADRB3* in sarcopenia or SO is scarce.

The potential roles of amino acid transporters are constantly investigated, and several hypotheses are suggested [125]. One regards the solute carrier 6, subfamily A of member 14 gene, which encodes SLC6A14—a Na+/Cl-transporter for amino acids (AA) [101]. The polymorphism of this gene, for example, SNP rs2011162; 22510 C>G, is associated with obesity [126,127]. SLC6A19 is another type of Na+-coupled transporter for neutral amino acids. It is involved in the intestinal absorption of dietary protein-derived amino acids and the renal reabsorption of circulating amino acids [128]. It also controls appetite by regulating tryptophan availability for serotonin synthesis [126]. Deleting *SLC6A19* protects against obesity and metabolic syndrome development and improves glycemic control. Thus, pharmacological inhibition of the transporter can benefit the control of metabolic parameters [129,130]. Recent findings show that SNP in the 3′UTR-region of its mRNA suppresses the transporter expression and causes metabolic disorders. In animal studies, such mutations predispose to obesity during a high-fat diet [101]. The same polymorphism is associated with reduced fat oxidation [131]. Unfortunately, data on SLC6A14’s role in sarcopenia are not available. 

PPARγ belongs to the nuclear receptor family and is activated by various fatty acid metabolites [132]. It is expressed mainly in the adipose tissue (both white and brown) and other organs, such as the liver, spleen, and large intestine [133]. PPARγ heterodimerizes with retinoid X receptor (RXR) in the nucleus, enabling gene transcription and expression regulation [132,133,134]. *PPARγ-2* gene acts as a regulator of metabolism and is strongly upregulated during adipogenesis. *PPARγ-2* is expressed in adipose tissue [92]. A study by Ren et al. demonstrated that *PPARγ-2* prevents lipotoxicity and prompts the increase of adipose tissue [133,135]. 

As a result of PPARγ stimulation, adipokines such as adiponectin, interleukin-6 (IL-6), leptin, or monocyte chemoattractant protein-1 (MCP-1) are released [133]. A missense mutation of *PPARγ* (rs1801282; C>G; Pro12Ala) plays an essential role in the pathogenesis of obesity. However, the impact of this polymorphism on obesity-related parameters is not clarified [136,137,138,139,140], which may result from the influence of diet and the genetic heterogeneity of various populations [141,142]. Postmenopausal overweight and obese women with Pro12Pro polymorphism of the *PPARγ* gene and higher lean body mass (>58% of body mass) have a bigger chance of recommended glucose levels compared to subjects with lower lean body mass [120]. 

A recent meta-analysis discovered worse metabolic parameters in obesity for the G allele carriers—including higher BMI, waist circumference, and total cholesterol [140]. Other common polymorphisms of the *PPARγ* gene are rs3856806; C>T; His447His, which is involved in an increased risk of obesity, coronary heart disease, or colorectal cancer, and rs1800571; Pro115Gln [137,143,144]. As SO is more common in older adults, there is a need to investigate the obesity and sarcopenia risk factors in this group. Some of them—like *PPARγ* mutation (rs1801282; C>G; Pro12Ala)—were described in elderly subjects to be a risk factor for increased obesity, especially in subjects with high carbohydrate intake with the co-presence of one rs9939609 allele of *FTO* gene [145].

Jones et al. discovered several genes responsible for low grip strength among 60+ patients. One is the growth/differentiation factor 5 (GDF5) protein. It concerns a 5′ untranslated region mutation of *GDF5*. GDF5 is a member of the transforming growth factor beta (TGF-β) family, impacting bone and joint development and the formation of osteoarthritis [85]. 

Genetic risk factors of sarcopenia were also assessed in the Korean population described by Park et al. Sarcopenia risk is associated with SNPs in fatty acid desaturase 2 (*FADS2*) (rs97384), *MYO10* (rs31574), *KCNQ5* (rs6453647), *DOCK5* (rs11135857), and low-density lipoprotein Receptor Related Protein 1B (*LRP1B*) (rs74659977) genes. However, it was assessed that the genetic impact might be masked by factors such as metabolic syndrome, high serum total cholesterol levels, and high grip strength. Lifestyle factors such as smoking, coffee, and alcohol intake did not interact with polygenic risk scores for sarcopenia. Exercise was highlighted as the only lifestyle change capable of overcoming genetic factors [66].

Common variants of *FADS2* rs1535 major alleles (A-alleles) are reported to be associated with higher BMI in children and adolescents [146]. *FADS2* polymorphisms affect the obesity risk in the adult population as well [147]. Moreover—some SNPs can manifest by enhancing circulating fatty acids, influencing predispositions of developing metabolic syndrome, or interacting with dietary non-enzymatic antioxidant capacity—thereby influencing cardio-metabolic risk factors [147,148]. Intronic or intragenic *FADS2* SNPs are associated with different levels of polyunsaturated fatty acids (PUFA) ω-6 and ω-3 in pregnant women [149]. LRP1B is a protein with an important role in lipid metabolism, including lipoprotein catabolism, and is linked to BMI and WHR in children [150]. These findings force further investigations on these genes and their possible link to SO.

Studies have shown that in women with co-occurring sarcopenia and obesity, mutations occurred in genes *ACTN3* and *MTHFR* [151]. However, it is worth noting that despite the identification of specific SNPs, the definition of SO adopted by the authors may vary, significantly affecting the study’s outcomes and potentially including polymorphisms whose association with SO, according to the current definition, is insufficient [151]. For instance, drawing upon the research by Khanal et al., for patients with sarcopenia diagnosed in compliance with the low percent of skeletal muscle mass (%SSM) definition, a positive correlation with SNPs in *FTO*, *ESR1*, and *NOS3* was found. However, females diagnosed with decreased SMI (skeletal muscle index) showed *TRHR* polymorphism only [110].

Another mechanism associated with SO is telomere shortening, analyzed by Goddard et al. Individuals with SO have significantly shorter telomeres than the control group [152]. Some genetic risk factors for obesity, sarcopenia, or SO are presented in Table 3.

### 5.2. Epigenetic Factors in Sarcopenic Obesity

The literature mentions various individual, environmental, and genetic risk factors for sarcopenia. Individual risk factors such as age [68,153], male gender [153], higher BMI [153], high monocyte level [153], and sleep duration exceeding 9 h [153] have been positively associated with SO. Advanced age (defined as ≥75 years old) is also associated with a reduction of motor function, resulting in muscle immobility, and predisposing to sarcopenia [68]. 

Another important risk factor of SO is food insecurity [67,154]. Food insecurity is poor food quality, lack of variety, and simultaneous excessive food consumption. Older people are generally more vulnerable to various factors such as functional disability or impairment, chronic health conditions, lower economic income, and a sedentary lifestyle. All those factors interact, leading to food insecurity [154]. Lowered protein intake, combined with higher calorie intake as well as insufficient vitamin and mineral intake, is part of food insecurity but is also defined as a modifiable risk factor for SO alone [66,68,154]. 

Sufficient fiber, zinc, vitamin D, calcium, and folate intake is associated with a lower risk of sarcopenia [66,67,68]. Sarcopenia is also associated with high serum glucose, total cholesterol, LDL-cholesterol, and triglyceride concentration [66]. The metabolic disorders related to inflammation, insulin resistance, and oxidative stress are proposed [68].

Recent research by Semenova et al. showed the association of sarcopenia with genetic risk factors. The study observed that the same polymorphisms contribute partially to both obesity and muscle loss, potentially explaining why most individuals affected by sarcopenia are also obese. According to the study, these identified polymorphisms are located in 73 genes with diverse functions, including those related to protein metabolism (*BCKDHB*, *BTRC*, *COMMD4*, *SERPINA1*, *WWP2*), carbohydrate metabolism (*ADPGK*, *CDKAL1*, *GIP*, *PRRC2A*), and lipid metabolism (*ADCY3*, *E2F3*, *HMGA2*, *MTCH2*, *NCOA1*, *NMT1*, *PPARδ*, immune system activity (*BTNL2*), myogenesis (*SFMBT1*), and intracellular transport (*GBF1*, *KIF1B*, *RIN3*, *SLC39A8*, *XPO4*) [67]. The same study identified risk alleles associated with sarcopenia. It was assessed that risk alleles are associated with traits such as tiredness, falls in the last 12 months, lowered physical activity, low mineral bone density, neuroticism, time spent watching television, smoking, and poor diet [67]. 

## 6. Conclusions

SO is a complex entity comprising immunogenetics as the major cause. The previous use of different definitions and criteria of SO components results in difficulties in estimating the disease’s prevalence, however researchers indicate a higher prevalence in older, as well as chronically ill patients. Both immune and genetic involvement is crucial. Increased adipose tissue leads to systemic inflammation, changes skeletal muscle metabolism, and results in SO and decreased oxidative capacity [47]. Pro-inflammatory cytokine synthesis is of significant importance in the pathophysiology of SO, further—many SNPs were discovered to play a crucial role in obesity (e.g., *MC4R* or *β3*) or sarcopenia separately; however, the exact genetic polymorphisms in SO are not well-established yet but remain suspected. More studies are needed to establish the exact genetic alterations leading to SO, leading to the possibilities of early prevention. Nevertheless, the issue of SO is often underestimated, and there is a need to enhance awareness among medical professionals.

## Figures and Tables

**Table 1:** Criteria of sarcopenia according to national consensuses.

Criteria of Sarcopenia		
Organization	Criteria	Reference
The New Mexico Elder Health Survey, 1993–1995 (1997) (1998)	Reference population: Rosetta study (*n* = 883) [18] Sarcopenia definition: an appendicular skeletal muscle mass (kg)/height^2^ (m^2^) less than two standard deviations below the mean of a young reference group.	[18,19]
International working group on sarcopenia—consensus of geriatricians and scientists (2011)	Sarcopenia definition: the age-associated loss of skeletal muscle mass and function. Diagnosis of sarcopenia: gait speed < than 1 m·s^−1^ objectively measured low muscle mass—appendicular mass relative to ht2 (M ≤ 7.23 kg/m^2^ and F ≤ 5.67 kg/m^2^)	[17]
The Foundation for the National Institutes of Health Biomarkers Consortium Sarcopenia Project, (2014)	1. Weakness Handgrip strength M < 26 kg; F < 16 kg Handgrip strength adjusted for BMI: M < 1.0 and F < 0.56 2. ALM: M < 19.75 kg and F < 15.02 kg 3. ALM adjusted for BMI: M < 0.789 and F < 0.512	[20]
The European Working Group on Sarcopenia in Older People 2 (EWGSOP2) (2019)	Criteria 1. Low muscle strength 2. Low muscle quantity or quality 3. Low physical performance Sarcopenia: probable—fulfilled criterion 1 diagnosed—fulfilled criterion 1 + documented criterion 2 severe—all criteria fulfilled	[2]
Australia and New Zealand. The Australian and New Zealand Society for Sarcopenia and Frailty Research (ANZSSFR) Sarcopenia, (2023)	1. Screening tools SARC-F may (is consistent with the EWGSOP2 algorithm) clinical suspicion (falls, weight loss, feeling weak, ↓ mobility 2. low muscle strength (handgrip strength, chair stand test) or clinical performance (decreed gait seed) 3. sarcopenia conformation: DXA, BIA, CT, MRI	[21]

↓—decrease; ALM = appendicular lean mass; BMI = body mass index; M—male; F—female; DXA—dual X-ray absorptiometry, BIA—bioelectrical impedance analysis; CT—computer tomography; MRI—magnetic resonance; EWGSOP2—the European Working Group on Sarcopenia in Older People.

**Table 3:** Genetic background of obesity, sarcopenia, and SO.

Disease and/or Its Definitions Used	Genetic Risk Factor	Studied Population; *n*	Definition Used/Conclusion	Citation
Obesity or impaired metabolic functions	*FTO* (rs9939609)	Korean adult women; *n* = 3335	- association of FTO with body fat markers	[108]
	*ADRB3*	adults; *n* = 265	- correlation of ADRB3 hypermethylation with overweight, obesity, higher waist-hip ratio, trans-fat intake and altered lipid profile	[115]
		schoolchildren; *n* = 72	Trp64Arg and Arg64Arg allele carriers present with higher LDL-c levels and lover maximal fat oxidation rates	[117]
Obesity or impaired metabolic functions (BMI ≥ 30 kg/m^2^)	*SLC6A14*	French-Caucasian adults; *n* = 1267, controls = 649	20649 C > T and 22510 C > G polymorphisms are associated with obesity	[127]
		Finnish men; *n* = 117, controls = 182	association of SLC6A14 polymorphisms with obesity	[126]
		*n* = 722 Europeans	22510 C > G allele reduces fasting fat oxidation	[131]
Sarcopenia	*FTO*	*n* = 2207	↓ LMI in 29 SNP of FTO	[109]
Sarcopenia %SMM sarcopenia definition used	*FTO* (rs9939609)	women > 60 years; *n* = 307	↑ sarcopenia risk	[110]
	*NOS3* (rs1799983)			
Sarcopenia	*GDF5* (rs143384)	Europeans > 60 yeas; *n* = 256.523	↓ grip strength	[85]
Sarcopenia SMI < 29.0% in men and SMI < 22.8% in women	*FADS2* (rs97384)	men and women > 50 years; *n* = 1599 controls = 23,391	- various factors might offset the genetic impact - exercise might overcome the genetic effect	[66]
	*MYO10* (rs3157)			
	*KCNQ5* (rs6453647)			
	*DOCK5* (rs11135857)			
	*LRP1B* (rs74659977)			
SO SMI < 6.76 kg/m^2^, HGS < 28.5 kg and BF% > 38%	*ACTN3* (rs1815739)	older women in England; *n* = 307	Sarcopenia in obese women	[151]
	*MTHFR* (rs1801131)			
	*MTHFR* (rs1537516)			

↓—decrease; ↑—increase; LMI—lean mass index (lean soft tissue/square of height); %SMM—skeletal muscle mass; SMI—skeletal muscle index (SMM/height2); HGS—handgrip strength; BF%—body fat%.
